# NMDA Receptors Regulate the Development of Neuronal Intrinsic Excitability through Cell-Autonomous Mechanisms

**DOI:** 10.3389/fncel.2017.00353

**Published:** 2017-11-07

**Authors:** Guoqiang Hou, Zhong-Wei Zhang

**Affiliations:** The Jackson Laboratory for Mammalian Genetics, Bar Harbor, ME, United States

**Keywords:** NMDA receptors, excitability, thalamus, development, brain slice electrophysiology, immunostaining, mTOR pathway, cell size

## Abstract

Maturation of neuronal and synaptic functions during early life is essential for the development of neuronal circuits and behaviors. In newborns synaptic transmission at excitatory synapses is primarily mediated by *N*-methyl-D-aspartate receptors (NMDARs), and NMDAR-mediated signaling plays an important role in synaptic maturation. Concomitant with synapse development, the intrinsic properties of neurons undergo dramatic changes during early life. However, little is known about the role of NMDARs in the development of intrinsic excitability. By using mosaic deletion of the obligatory GluN1 subunit of NMDARs in the thalamus of newborn mice, we showed that NMDARs regulate neuronal excitability during postnatal development. Compared with neighboring control neurons, neurons lacking NMDARs exhibit hyperexcitability and this effect is present throughout early life. Morphological analyses show that thalamic neurons without NMDARs have smaller soma size and fewer dendritic branches. Deletion of NMDARs causes a reduction of hyperpolarization-activated cation (HCN) channel function in thalamic neurons, and pharmacologically blocking HCN channels in wild type neurons mimics the effects of GluN1 deletion on intrinsic excitability. Deletion of GluN1 down-regulated mechanistic target of rapamycin (mTOR) signaling in thalamic neurons, and mosaic deletion of mTOR recapitulated the effects of GluN1 deletion. Our results demonstrate that NMDARs regulate intrinsic excitability and morphology of thalamic neurons through cell autonomous mechanisms that implicate mTOR signaling.

## Introduction

Activation of *N*-methyl-D-aspartate receptors (NMDARs) by glutamate at excitatory synapses opens cation-selective channels with high permeability to Ca^2+^ ions and generates excitatory postsynaptic currents (EPSCs) with a slow time course. NMDARs have critical functions in the development and plasticity of glutamatergic synapses (Malinow and Malenka, 2002; Song and Huganir, 2002). In the brain of newborns, glutamatergic synapses contain few or no α-amino-3-hydroxy-5-methyl-4-isoxazolepropionic acid receptors (AMPARs), and synaptic transmission is mediated primarily by NMDARs (Isaac et al., 1995, 1997; Liao et al., 1995). Synaptic maturation is associated with an increase of AMPARs at the synapse, and this developmental change of AMPARs is regulated by NMDARs (Liao et al., 1999; Adesnik et al., 2008; Zhang et al., 2013). NMDARs are also required for pruning of redundant synaptic inputs during development (Ruthazer et al., 2003; Zhang et al., 2013).

Together with synaptic maturation, the intrinsic membrane properties of neurons undergo dramatic changes during early life. Cortical and thalamic neurons show negative shifts in resting potential and action potential threshold, an acceleration of action potential kinetics, and a reduction of input resistance during the first 3 weeks after birth (Ramoa and McCormick, 1994; Zhou and Hablitz, 1996; Franceschetti et al., 1998; Zhu, 2000; Zhang, 2004). Neuronal activity plays an important role in the development of intrinsic excitability (Spitzer and Ribera, 1998; Spitzer, 2006). In embryonic *Xenopus* spinal neurons, transient elevations of intracellular Ca^2+^ caused by action potentials serve as a key messenger for the expression of ion channels (Gu and Spitzer, 1995). Visual stimulation in *Xenopus* tadpoles increases intrinsic excitability of optical tectal neurons (Aizenman et al., 2003). Activation of NMDARs causes Ca^2+^ influx either directly through the channels or indirectly by triggering action potentials. However, the role of NMDARs in the development of intrinsic excitability of neurons is poorly understood.

Previous studies have shown that NMDARs regulate the development of neuronal morphology. In *Xenopus* embryos, pharmacological block of NMDARs inhibited activity-dependent dendritic arborization of optical tectal neurons (Rajan and Cline, 1998; Sin et al., 2002). The role of NMDARs in dendritic development has also been studied using knockout mice. NMDARs are generally heterotetramers consisting of two obligatory GluN1 subunits and a combination of two GluN2 subunits (GluN2A-D) (Cull-Candy et al., 2001). Selective deletion of GluN1 from all excitatory neurons in the cortex disrupted dendritic patterning of layer 4 neurons (Datwani et al., 2002). Similar results have been obtained with mosaic deletion of GluN2B, the primary GluN2 subunit in the developing brain (Espinosa et al., 2009), indicating that the effect of NMDARs on dendritic patterning is cell-autonomous.

The hyperpolarization-activated current (*I*_h_) plays important roles in the regulation of neuronal intrinsic excitability during development and in adult (Robinson and Siegelbaum, 2003; Bender and Baram, 2008; Biel et al., 2009; Noam et al., 2011). *I*_h_ is a non-inactivating Na^+^/K^+^ conductance activated by hyperpolarization at membrane potentials close to the resting membrane potential of neurons. Because of its unique properties, *I*_h_ has an essential role in the regulation of resting membrane potential, input resistance, and other voltage-dependent mechanisms of neurons. *I*_h_-conducting HCN channels shows temporal and spatial patterns of expression in the brain (Moosmang et al., 1999; Bender et al., 2001; Notomi and Shigemoto, 2004). Developmental changes of HCN channel expression have been implicated in the maturation of network activity in the hippocampus (Bender et al., 2005). In thalamus, the upregulation of *I*_h_ in thalamic neurons during early life is associated with increased expression of HCN1, 2 and 4 (Kanyshkova et al., 2009).

In the present study, we directly address the role of NMDARs in the development of intrinsic excitability of neurons in the thalamus. Because deletion of NMDARs from a large number of neurons can cause indirect effects due to changes in network activity, we use a genetic mosaic method to delete GluN1 from a fraction of neurons in thalamus, allowing us to assess cell-autonomous functions of NMDARs. We find that NMDARs are required for the maturation of intrinsic excitability and morphology of thalamic neurons. This function of NMDARs is cell autonomous and implicates mTOR signaling and HCN channels.

## Materials and Methods

### Animals

The conditional allele of *Grin1* (*Grin1^fl^*, JAX #018825, The Jackson Laboratory, Bar Harbor, ME, United States) (Zhang et al., 2013), SERT-Cre [Tg(*Slc6a4*-cre)ET124Gsat, Mutant Mouse Resource and Research Center (MMRRC)] (Gong et al., 2007), Rosa26-tdTomato reporter (*Rosa*^*Ai*14^, JAX #007914, The Jackson Laboratory) (Madisen et al., 2010), conditional allele of *Mtor* (*Mtor^fl^*, JAX #011009, The Jackson Laboratory) (Risson et al., 2009), *Gria3* knockout (B6-129P2-*Gria3*^tm1-Dgen^, MMRRC), and *Gria4* knockout (B6-129P2-*Gria4*^tm1-Dgen^, MMRRC) were housed in the Research Facility at The Jackson Laboratory. All the strains were on a C57BL/6J background. Both male and female mice were used in the experiments. Genotyping were determined by PCR using tail genomic DNA and allele-specific primers. For the SERT-Cre strain, the primers were forward 5′-CAACAGAGCTCTCAGTCTTGTC-3′ and reverse 5′-TGGTGTACGGTCAGTAAATTGG-3′. For *Gria3* knockout strain, the primers were wild type forward 5′-GGGTGGGATTAGATAAATGCCTGCTCT-3′, mutant forward 5′-GGTCACGAGGTTCTTCATTGTTGTC-3′, and common reverse 5′-AGCTGATATAGCTGTTGCTCCACTC-3′. For *Gria4* knockout strain, the primers were forward 5′-TGTCACAGCAAAACTGTTGGCAGT-3′, and reverse 5′-GGGTGG GATTAGATAAATGCCGCTC-3′. Primer sequences for all the other strains can be found at the website of The Jackson Laboratory. The Jackson Laboratory Animal Care and Use Committee approved all animal protocols.

### Electrophysiology

Mice were anesthetized with tribromoethanol and decapitated. Brains were dissected quickly and sagittal sections 300 μm thick were prepared with a vibratome (Leica VT1200) and kept in artificial cerebral spinal fluid (ACSF) containing (in mM): 124 NaCl, 3.0 KCl, 1.5 CaCl_2_, 1.3 MgCl_2_, 1.0 NaH_2_PO_4_, 26 NaHCO_3_, and 20 glucose, saturated with 95% O_2_ and 5% CO_2_ at room temperature (21–23°C). We used 1.5 mM Ca^2+^ in ACSF because a previous study showed that the concentration of Ca^2+^ in the cerebral spinal of young rats is about 1.5 mM (Jones and Keep, 1988).

For patch clamp recording, a slice was transferred to a submersion type chamber where it was continuously perfused with ACSF saturated with 95% O_2_ and 5% CO_2_ at 30–32°C. Neurons in the VPm were viewed with a 40x objective and Cre^+^ neurons were identified by epifluorescence of tdTomato. Whole-cell recordings were obtained from the soma with a Multiclamp 700B amplifier (Molecular Devices, Sunnyvale, CA, United States). The pipette solution contained (in mM): 120 K-gluconate, 10 KCl, 4 ATP-Mg, 0.3 GTP-Na, 0.5 EGTA, and 10 HEPES (pH 7.2, 270–280 mOsm with sucrose). For current clamp recording, the series resistance (R_s_) was fully compensated. For voltage clamp recording, R_s_, usually between 8 and 12 Mω, was continuously monitored but not compensated. Data was discarded when R_s_ changed by more than 30% during the experiment. Experiments were conducted using AxoGraph X (AxoGraph Scientific). Data were filtered at 4 kHz and digitized at 20 kHz. Data analysis was performed using AxoGraph X.

Intrinsic properties of neurons were analyzed using methods described previously (Zhang, 2004; Zhang and Arsenault, 2005). Resting membrane potentials were measured within 20 s of break in. Input resistance and time constant were calculated from voltage responses to 600-ms current steps of -100 pA. For the analyses of AP rheobase and F-I curves, current steps of 1 s in duration were applied once every 10 s, with increments of 10 or 20 pA. To minimize the effect of resting potential variation on rheobase and F-I curve, cells were held at -60 mV between current steps. In some experiments, synaptic blockers picrotoxin, kynurenic acid, and 6,7-Dinitroquinoxaline-2,3-dione (DNQX; all from Tocris) were applied through bath perfusion.

Voltage-dependency of *I*_h_ activation was analyzed using a voltage-clamp protocol similar to that described in a previous study (Kanyshkova et al., 2009). This protocol takes into consideration voltage-dependent changes in the onset rate of *I*_h_. Initially held at -50 mV, the membrane potential of the cell was stepped to increasingly hyperpolarizing levels for several seconds to reach steady-state activation, then to -100 mV. The activation curve of *I*_h_ was established by measuring the tail currents at 40 ms after stepping to -100 mV. The normalized *I*_h_, *p(V)*, was calculated using the equation:

$$p(V)=(I-I_{\min})/\left(I_{\max}-I_{\min}\right)$$

where *I_min_* and *I_max_* are the amplitudes of tail currents for pre-pulses at -50 and -150 mV, respectively. The activation curves of *I*_h_ were fitted to a Boltzmann equation:

$$p(V)=1/(1+\exp\left((V-V_{h})/k\right)),$$

where *V*_h_ is the membrane potential of half-maximal activation, and *k* is the slope factor.

Recordings of EPSCs at VPm relay synapses were performed as described previously (Arsenault and Zhang, 2006; Wang and Zhang, 2008). The pipette solution contained (in mM): 110 Cs methylsulfate, 20 TEA-Cl, 15 CsCl, 4 ATP-Mg, 0.3 GTP, 0.5 EGTA, 10 HEPES, 4.0 QX-314 and 1.0 spermine (pH 7.2, 270–280 mmol kg^-1^ with sucrose). A concentric bipolar electrode (FHC, Bowdoin, ME, United States) was placed in the medial lemniscus, and stimuli were applied at 0.1 Hz. GABAergic transmission was blocked by picrotoxin (100 μM) in the bath.

### Single Cell Labeling and Reconstruction

Neurons were labeled during whole-cell recording with 0.3% biocytin in the patch pipette. Slices were fixed in 4% paraformaldehyde in 0.1 M phosphate buffer (PB) overnight and processed as described previously (Zhang and Deschenes, 1997; Zhang, 2004). Briefly, slices were treated with 0.3% H_2_O_2_ in 10% MeOH in 0.1 M PB to inactivate endogenous peroxidase. After wash, slices were processed for biocytin histochemistry using the ABC Elite Standard Kit (Vector Laboratories, Burlingame, CA, United States), 3,3-diaminobenzidine, and nickel ammonium sulfate. Cells were reconstructed and analyzed with the Neurolucida System (MicroBrightfield, Williston, VT, United States).

### Immunostaining

Mice were anesthetized with tribromoethanol and perfused with 4% paraformaldehyde in 0.1 M PB. Brains were post fixed over night. Sagittal sections of 70 μm thickness were cut on a vibratome. Sections were incubated with primary antibodies for 48 h at 4°C. The primary antibodies were NeuN (Millipore, MAB377, mouse, 1:1000), p-S6 (Ser240/244) (Cell Signaling, Beverly, MA, United States #5364, rabbit, 1:1000), p-Akt (Thr308) (Cell Signaling, #2965, rabbit, 1:1000), and RFP (Rockland, Limerick, PA, United States #200-301-379, mouse, 1:1000). For p-Akt staining, sections were pretreated with pepsin (Sigma, 0.5 mg/ml in 0.2 N HCl) for 5 min at 37°C before incubation with primary antibody. Secondary antibodies were conjugated with Alexa fluor (Life Technologies, 1:500). Sections were mounted in ProLong Diamond (Life Technologies). Confocal stacks were taken with a 63x (NA 1.4) objective on a Leica SP5 microscope. The pixel size was 80.2 nm. The z-step size was 130 nm and each stack had 10 steps.

Data were analyzed using ImageJ. Changes in cell body size were quantified by measuring soma area. Confocal stacks of thalamic neurons immunostained for NeuN were z-projected at maximum intensity. Cre^+^ neurons were identified by the expression of the reporter protein tdTomato. Cell bodies of individual neurons were outlined using the polygon selection tool and soma area was analyzed. The same method was used to quantify immunofluorescence of p-Akt and p-S6. The mean fluorescence intensity of immunofluorescence staining was measured for the soma.

### Statistical Analysis

Statistical analyses were performed with JMP software (SAS, Cary, NC, United States). Data were presented either as median with interquartile range and the minimum and maximum (box and whisker) or as mean ± SEM. Statistical differences were determined using non-parametric Wilcoxon Rank Sum Test (two groups) or Krustal–Wallis Test (three or more groups). *P*-values were provided in the results or figure legends.

## Results

### Mosaic Deletion Demonstrates Cell-Autonomous Role of NMDA Receptors in Regulating Intrinsic Excitability

**FIGURE 1:**
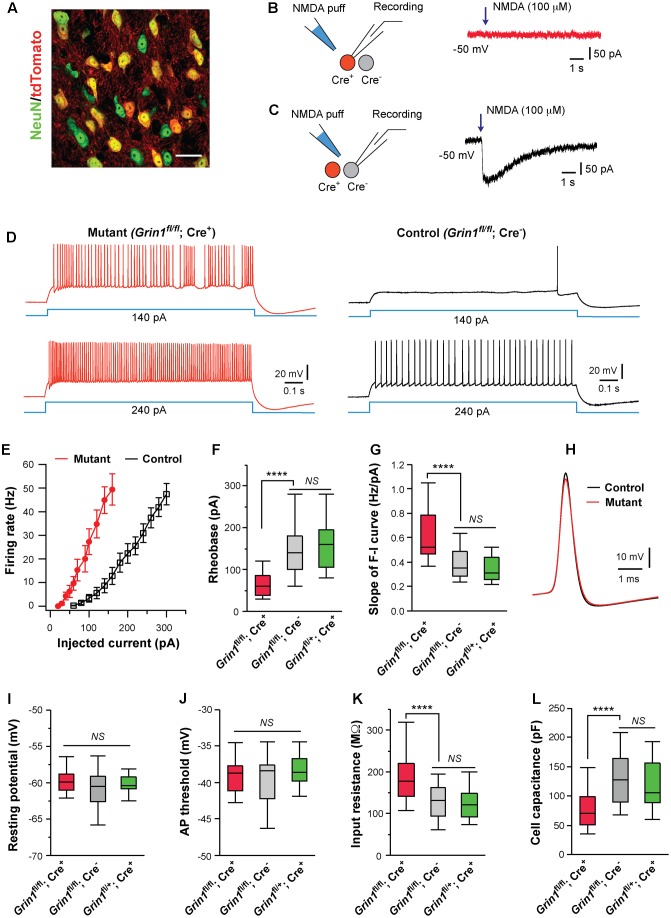
Cell autonomous effects of NMDAR deletion on intrinsic excitability of thalamic relay neurons. **(A)** Confocal image of VPm neurons in a SERT-Cre; *Rosa*^*Ai*14^ mouse at P14. The section was immunostained for NeuN (green). Scale bar: 30 μm. **(B,C)** Current responses to pressure applications of NMDA (100 μM, 200 ms puff) in a mutant neuron (*Grin1^fl/fl^*; Cre^+^) and a control neuron (*Grin1^fl/fl^*; Cre^-^), respectively, in a SERT-Cre; *Grin1^fl/fl^*; *Rosa*^*Ai*14^ mouse at P15. **(D)** Firing patterns of a mutant neuron (traces in red on the left) and a control neuron (traces in black on the right) in a SERT-Cre; *Grin1^fl/fl^*; *Rosa*^*Ai*14^ mouse at P15. The membrane potential of the cells was adjusted to –60 mV before each current step. **(E)** The F-I curves for control and mutant neurons recorded from SERT-Cre; *Grin1^fl/fl^*; *Rosa*^*Ai*14^ mice at P14-16. **(F)** Rheobase of mutant (*Grin1^fl/fl^*; Cre^+^), control (*Grin1^fl/fl^*; Cre^-^), and Cre^+^ control neurons (*Grin1^fl/+^*; Cre^+^) recorded at P14-16 (^∗∗∗∗^*p* < 0.0001; *NS*, *p* = 0.35, Wilcoxon test). **(G)** Slope of the F-I curve of the three groups (^∗∗∗∗^*p* < 0.0001; *NS*, *p* = 0.14, Wilcoxon test). **(H)** Examples of action potentials from mutant (red) and control (black) neurons. **(I)** Resting potential of the three groups (*NS*, *p* = 0.53, Wilcoxon test). **(J)** AP threshold of the three groups (*NS*, *p* = 0.34, Wilcoxon test). **(K)** Input resistance of the three groups (^∗∗∗∗^*p* < 0.0001; *NS*, *p* = 0.11, Wilcoxon test). **(L)** Whole-cell capacitance of the three groups (^∗∗∗∗^*p* < 0.0001; *NS*, *p* = 0.61, Wilcoxon test). Data in **(F,G,I-L)** are presented as median with interquartile range and the minimum and maximum (box and whisker plot). Data were collected from 22 mutant neurons (*Grin1^fl/fl^*; Cre^+^) and 27 control neurons (*Grin1^fl/fl^*; Cre^-^) from 13 mice, and 19 Cre^+^ control neurons (*Grin1^fl/+^*; Cre^+^) from 7 mice.

*N*-methyl-D-aspartate receptors are ubiquitously expressed in the brain during development. Pharmacological block or genetic deletion of NMDARs from a large number of neurons disrupts activities of neuronal circuits. To distinguish cell-autonomous vs. non-autonomous effects, we performed mosaic deletion of NMDARs in the thalamus of newborn mice by using the conditional allele of the NMDAR subunit GluN1 (*Grin1^fl^*) (Zhang et al., 2013) and the transgenic serotonin transporter Cre strain (SERT-Cre) (Gong et al., 2007; Zhang et al., 2013). Our previous study showed that in SERT-Cre; *Grin1^fl/fl^* mice, about 50% of VPm neurons express Cre and lack NMDARs, and the loss of NMDAR function occurs between P7 and P10 (Zhang et al., 2013). The expression of Cre in VPm neurons was random. To identify neurons with or without Cre expression, we used the *Rosa26*-tdTomato reporter (*Rosa*^*Ai*14^) (Madisen et al., 2010) to express the fluorescent marker tdTomato in Cre^+^ neurons (**Figure 1A**). We first recorded VPm neurons in acute slices obtained from SERT-Cre; *Grin1^fl/fl^*; *Rosa*^*Ai*14^ mice at P14-16, the age at which VPm neurons reach maturity in synaptic connectivity (Arsenault and Zhang, 2006; Wang et al., 2011). We applied NMDA to the recorded neurons to confirm the absence or presence of NMDARs (**Figures 1B,C**). Intrinsic excitability was analyzed with whole-cell patch clamp recording.

Deletion of NMDARs dramatically altered excitability of VPm neurons at P14-16 (**Figures 1D,E**). Compared with neighboring control neurons with NMDARs (*Grin1^fl/fl^*; Cre^-^), mutant neurons deficient of NMDARs (*Grin1^fl/fl^*; Cre^+^) had lower rheobases for firing action potential (AP), and steeper slopes of the frequency to current (F-I) curves (**Figures 1F,G**). Mutant neurons showed a small reduction in AP amplitude (**Figure 1H**; 67.5 ± 1.1, *n* = 22 for mutant vs. 71.4 ± 1.0 mV, *n* = 27 for control; ^∗∗^*p* = 0.005, Wilcoxon test) and a small increase in AP half width (0.42 ± 0.01 vs. 0.37 ± 0.01 ms; ^∗∗^*p* = 0.002, Wilcoxon test), but no change in AP rise time (0.13 ± 0.01 ms vs. 0.12 ± 0.01 ms; *p* = 0.12, Wilcoxon test). There was no difference between mutant and control neurons in after hyperpolarization (-9.2 ± 0.5 mV for mutant vs. -9.7 ± 0.4 mV for control neurons, *p* = 0.31, Wilcoxon test). There was no significant difference between mutant and control neurons in resting membrane potential, or AP threshold (**Figures 1I,J**). Mutant neurons had significantly higher input resistance and lower whole-cell capacitance than control neurons (**Figures 1K,L**). Mutant neurons showed a small reduction in the time constant (14.0 ± 0.1 ms, *n* = 22 for mutant vs. 16.3 ± 0.1 ms, *n* = 27 for control; ^∗^*p* = 0.012, Wilcoxon test).

Previous studies have shown that SERT is transiently expressed in all VPm neurons during early life (Lebrand et al., 1998; Narboux-Neme et al., 2008), so it is unclear why only a fraction of VPm neurons in the SERT-Cre strain express Cre. To exclude the possibility that SERT-Cre is expressed in a subpopulation of VPm neurons with distinct intrinsic excitability, we recorded Cre^+^ neurons in the VPm of SERT-Cre; *Grin1^fl/+^*; *Rosa*^*Ai*14^ mice at P14-16. All Cre^+^ neurons (*Grin1^fl/+^*; Cre^+^) in these *Grin1* heterozygous mice had normal NMDAR function. We did not find any difference in intrinsic excitability between *Grin1^fl/+^*; Cre^+^ neurons (**Figures 1F,G,I–L**, in green) and *Grin1^fl/fl^*; Cre^-^ neurons, indicating that in the presence of NMDARs, Cre^+^ and Cre^-^ neurons are indistinguishable in membrane property and excitability. Together, these findings suggest that the deletion of NMDARs alters excitability of VPm neurons through cell-autonomous mechanisms.

The experiments described above were conducted in the absence of any synaptic blockers. Deletion of NMDARs may alter spontaneous synaptic responses, which in turn, affect neuronal excitability. To examine this possibility, we performed recordings in the presence of glutamatergic blockers DNQX and kynurenic acid, and the GABAergic blocker picrotoxin. The differences in excitability between mutant and control neurons persisted in the presence of these synaptic blockers (Supplementary Figures S1A–E). These results show that the effects of NMDAR deletion on excitability in thalamic neurons are independent of spontaneous synaptic transmission.

**FIGURE 2:**
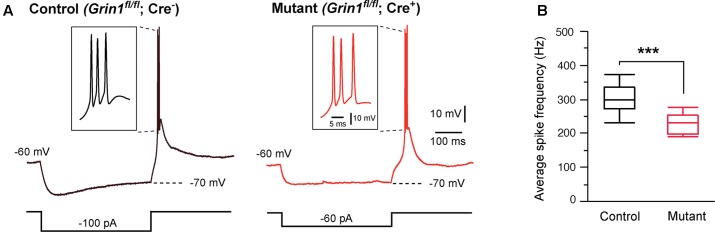
Effects of *Grin1* deletion on burst firing of thalamic neurons. **(A)** Rebound bursts recorded from a control and a *Grin1* mutant neuron at P15. The number of action potentials in a burst was not different between mutant (3.1 ± 0.3, *n* = 11 cells) and control (2.8 ± 0.3, *n* = 12 cells; *p* = 0.39, Wilcoxon test). **(B)** The average firing frequency of bursts recorded from mutant (229 ± 9 Hz, *n* = 11) and control (308 ± 16 Hz, *n* = 12) neurons (^∗∗∗^*p* = 0.0007, Wilcoxon test). Data were collected from 8 mice aged P14–16.

Thalamic relay neurons fire bursts of APs when stimulated from hyperpolarized membrane potentials. We analyzed rebound bursts following hyperpolarizing current steps. Both mutant and control neurons showed rebound bursts when hyperpolarized to -70 mV (**Figure 2A**). There was no difference in the number of APs in a burst between mutant (3.1 ± 0.3, *n* = 11 cells) and control (2.8 ± 0.3, *n* = 12 cells; *p* = 0.39, Wilcoxon test). However, the average firing frequency was significantly reduced in mutant neurons (**Figure 2B**).

**FIGURE 3:**
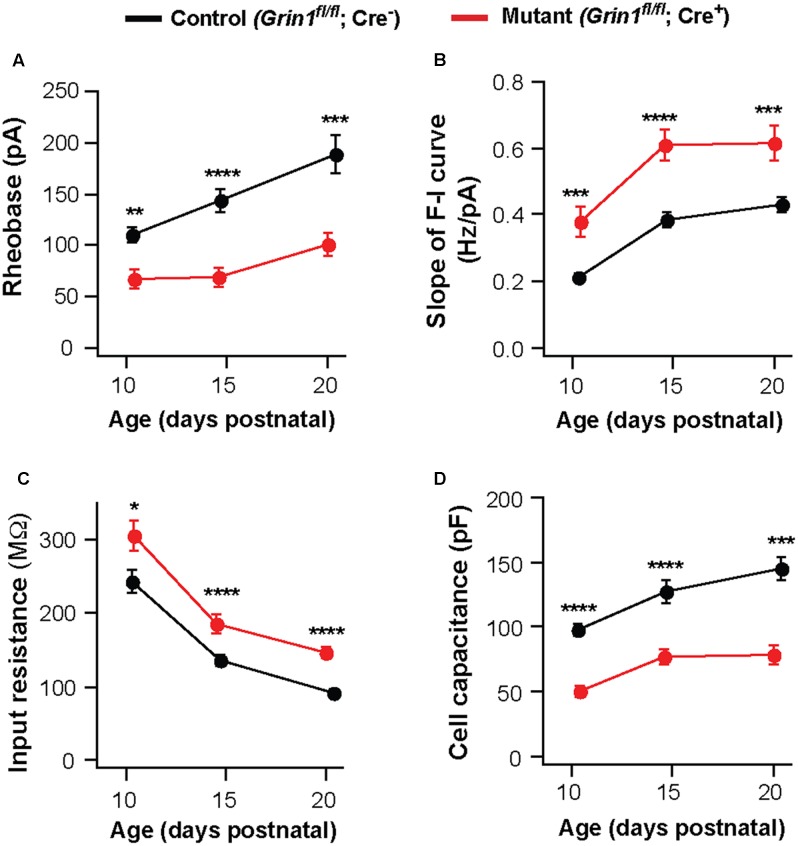
Persistent effects of NMDAR deletion on intrinsic excitability during early life. Data were collected from mutant (red) and control (black) thalamic neurons from SERT-Cre; *Grin1^fl/fl^*; *Rosa*^*Ai*14^ mice at P10–11, P14–16, and P20–21. **(A)** Rheobase of mutant and control neurons (P10–11, ^∗∗^*p* = 0.003; P14–16, ^∗∗∗∗^*p* < 0.0001; P20–21, ^∗∗∗^*p* < 0.001, Wilcoxon test). **(B)** Slope of the F-I curve of mutant and control neurons (P10–11, ^∗∗∗^*p* < 0.001; P14–16, ^∗∗∗∗^*p* < 0.0001; P20–21, ^∗∗∗^*p* < 0.001, Wilcoxon test). **(C)** Input resistance of mutant and control neurons (P10–11, ^∗^*p* = 0.017; P14–16, ^∗∗∗∗^*p* < 0.0001; P20–21, ^∗∗∗∗^*p* < 0.001, Wilcoxon test). **(D)** Whole-cell capacitance of mutant and control neurons (P10-11, ^∗∗∗∗^*p* < 0.0001; P14–16, ^∗∗∗∗^*p* < 0.0001; P20–21, ^∗∗∗^*p* < 0.001, Wilcoxon test). P10–11 data were obtained from 20 control and 15 mutant neurons in 9 mice. P20–21 data were obtained from 15 control and 16 mutant neurons in 6 mice. Data for the P14–16 group were the same as in **Figure 1**. Data were presented as mean ± SEM.

Deletion of NMDARs may cause a delay in the maturation of neuronal excitability. To examine this possibility, we analyzed VPm neurons at P10-11 and P20-21. The effects of NMDAR deletion on excitability and membrane properties were already present at P10-11. Neurons deficient of NMDARs showed a decrease in rheobase (**Figure 3A**) and an increase in the slope of the F-I curve (**Figure 3B**), together with an increase in input resistance (**Figure 3C**) and a decrease in whole-cell capacitance (**Figure 3D**). These effects are comparable to those observed at P14-16 and P20-21 (**Figures 3A–D**). These results suggest that NMDARs are required for postnatal development of intrinsic excitability in thalamic neurons.

**FIGURE 4:**
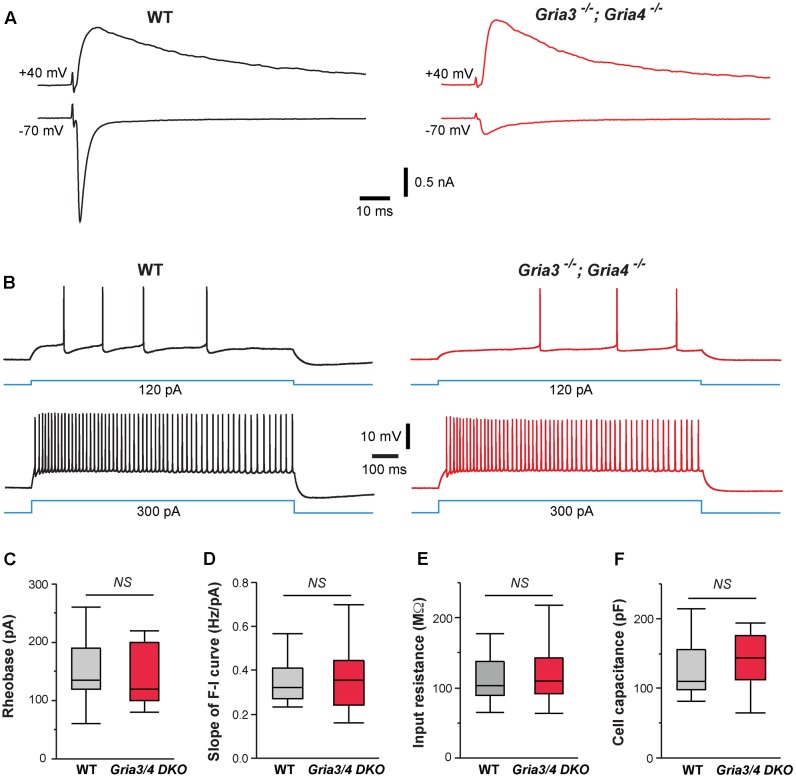
Deletion of GluA3 and GluA4 in thalamic neurons has no effect on intrinsic excitability. **(A)** EPSCs recorded at –70 and +40 mV from a WT (traces in black on the left) and a *Gria3/Gria4* double mutant neuron (traces in red on the right) recorded at P14. EPSCs were evoked by stimuli applied to the medial lemniscus as shown in Supplementary Figure S2A. Both neurons showed all-or-none responses. **(B)** Firing patterns of a WT (traces in black) and a *Gria3/Gria4* double mutant neuron (traces in red) recorded at P15. **(C)** Rheobase (*NS*, *p* = 0.60, Wilcoxon test), **(D)** Slope of F-I curve (*NS*, *p* = 0.63, Wilcoxon test), **(E)** Input resistance (*NS*, *p* = 0.65, Wilcoxon test), and **(F)** Cell capacitance (*NS*, *p* = 0.83, Wilcoxon test) of WT (*n* = 21 cells from 5 mice) and *Gria3/Gria4* double mutant neurons (*Gria3/4* DKO, *n* = 23 cells from 3 mice) recorded at P14-16.

Our previous study showed that deletion of NMDARs down-regulates synaptic AMPARs in VPm neurons (Zhang et al., 2013). The amplitudes of AMPAR-mediated EPSCs were reduced by 80% in VPm neurons deficient of NMDARs. To determine whether the effects of NMDAR deletion on intrinsic excitability are mediated by changes in AMPAR function, we used mice deficient of GluA3 (*Gria3*) and GluA4 (*Gria4*), two major AMPAR subunits for the thalamus. We have previously shown that deletion of both *Gria3* and *Gria4* reduces AMPAR-mediated EPSCs by 80% in VPm neurons without any changes in NMDAR-mediated EPSCs (Wang et al., 2011). Consistent with our previous finding, AMPAR-mediated EPSCs were dramatically reduced in VPm neurons from mice deficient of GluA3 and GluA4 (**Figure 4A**). However, intrinsic excitability of VPm neurons was not affected by the deletion of both GluA3 and GluA4 (**Figure 4B**). There was no difference between mutant and control neurons in the rheobase, slope of the F-I curve, input resistance, or whole-cell capacitance (**Figures 4C–F**). These findings suggest that the effects of NMDAR deletion on intrinsic excitability are not caused by the reduction in AMPAR functions.

### NMDARs Regulate Cell Size and Dendritic Morphology

**FIGURE 5:**
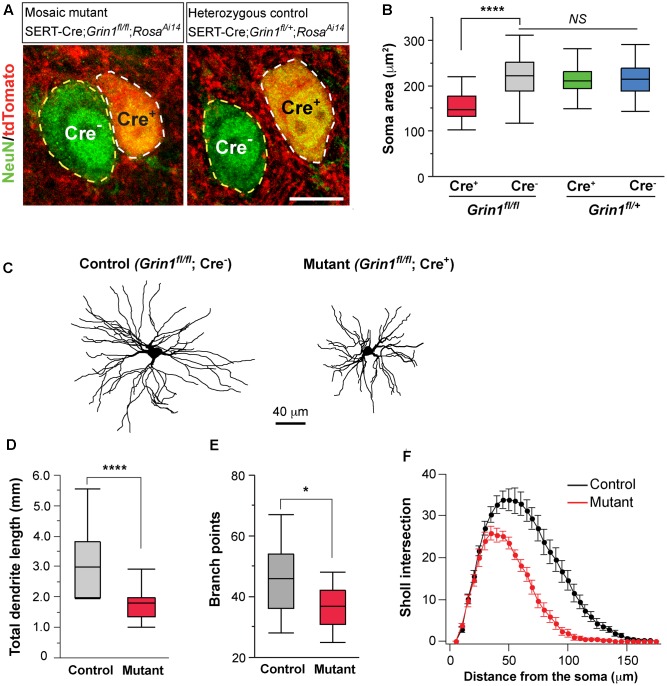
Deletion of NMDARs in thalamic neurons disrupts neuronal morphology. **(A)** Confocal images of VPm neurons in sections from a homozygous *Grin1* mouse (Mosaic mutant: SERT-Cre; *Grin1^fl/fl^*; *Rosa*^*Ai*14^) (image on the left) and a heterozygous *Grin1* mouse (heterozygous control: SERT-Cre; *Grin1^fl/+^*; *Rosa*^*Ai*14^) (image on the right) at P16. Sections were immunostained for NeuN. Cre^+^ neurons were identified by the presence of tdTomato at cell body. Soma of neurons are outlined with dash lines. Scale bar: 10 μm. **(B)** Cell body area of Cre^+^ and Cre^-^ neurons in homozygous *Grin1* (SERT-Cre; *Grin1^fl/fl^*; *Rosa*^*Ai*14^) and heterozygous *Grin1* (SERT-Cre; *Grin1^fl/+^*; *Rosa*^*Ai*14^) mice at P16. Data for each group were obtained from 80 to 90 cells from 3 mice. ^∗∗∗∗^*p* < 0.0001; *NS*, *p* = 0.38, Wilcoxon test. **(C)** Reconstruction of control (*Grin1^fl/fl^*; Cre^-^) and mutant (*Grin1^fl/fl^*; Cre^+^) neurons labeled at P16. **(D)** Total dendrite length of control and *Grin1* mutant neurons. ^∗∗∗∗^*p* < 0.0001, Wilcoxon test. **(E)** Number of branch points of control and mutant neurons. ^∗^*p* = 0.037, Wilcoxon test. **(F)** Distributions of Sholl intersections for control and *Grin1* mutant neurons. ^∗∗∗∗^ p < 0.0001, One-Way ANOVA with repeated measurements. Data presented in **(D,E)**, and **(F)** were obtained from 18 *Grin1* mutant and 15 control cells from 5 mice aged P14–16.

Neuronal morphology is an important determinant of cellular excitability. The reduction of whole-cell capacitance in NMDAR-deficient neurons indicates a decrease in cell size. We further examined the effect of NMDAR deletion on cell body size at P16-18. NeuN staining was used to label neuronal somata and soma area was quantified. In the VPm of mosaic mutant mice (SERT-Cre; *Grin1^fl/fl^*; *Rosa*^*Ai*14^), Cre^+^ mutant neurons (**Figure 5A**, left panel, cell body encircled by while dash line) had smaller soma size than neighboring Cre^-^ control neurons (cell body encircled by yellow dash line) (**Figure 5B**, red vs. gray). To determine whether SERT-Cre expression by itself may affect soma size, we also analyzed soma area in the VPm of heterozygous control mice (SERT-Cre; *Grin1^fl/+^*; *Rosa*^*Ai*14^) at P16-18 (**Figure 5A**, right panel). There was no difference in soma area between Cre^+^ and Cre^-^ in *Grin1* heterozygous mice (**Figure 5B**, green vs. blue). Importantly, there was no difference between control neurons (Cre^-^) in *Grin1* homozygous mice and neurons (Cre^+^ or Cre^-^) in *Grin1* heterozygous mice (**Figure 5B**). These results demonstrate that NMDARs regulate cell size in the thalamus through cell-autonomous mechanisms.

To determine the effects of NMDAR deletion on dendritic morphology, we labeled VPm neurons from SERT-Cre; *Grin1^fl/fl^*; *Rosa*^*Ai*14^ mice at P14-16 with biocytin in the whole-cell recording pipettes. Dendritic branches of individual VPm neurons were reconstructed and analyzed (**Figure 5C**). The total dendritic length was significantly shorter in mutant neurons (**Figure 5D**). Mutant neurons also had few dendritic branches than control neurons (**Figure 5E**). We performed Sholl analysis of dendritic morphology. The distribution of Sholl intersections was significantly different between mutant and control neurons (**Figure 5F**). The majority of Sholl intersections were much closer to the cell body for mutant than control neurons. These results suggest that NMDAR signaling regulates the growth of dendritic branches of thalamic neurons.

### HCN Channels Are Implicated in the Regulation of Intrinsic Excitability by NMDARs

**FIGURE 6:**
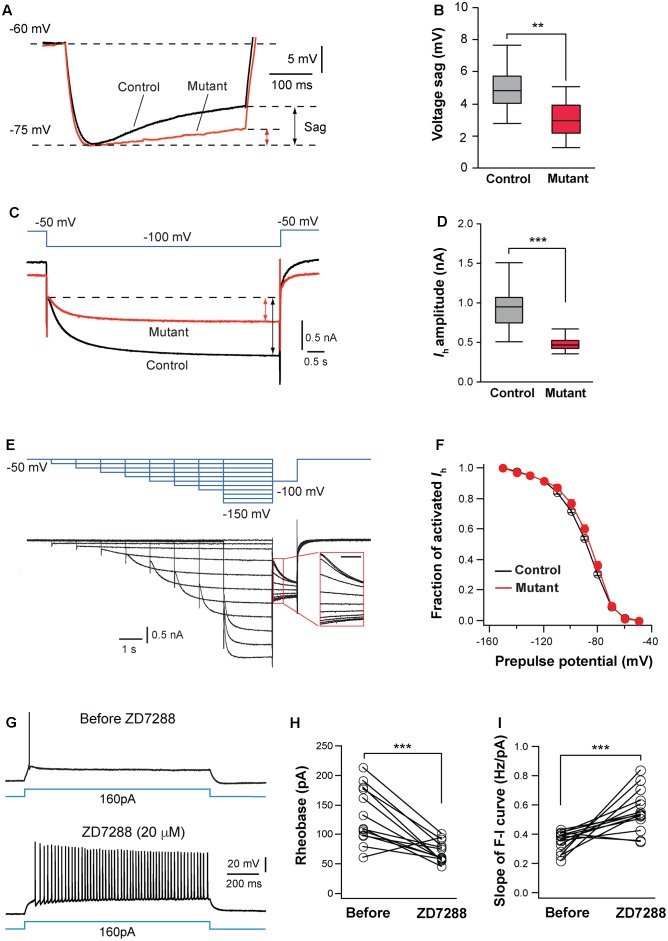
Hyperpolarization-activated cation channels are implicated in hyperexcitation caused by NMDAR deletion. **(A)** Voltage sag generated by *I*_h_ in a control (black) and mutant (red) in SERT-Cre; *Grin1^fl/fl^*; *Rosa*^*Ai*14^ mice at P15. Cells were hyperpolarized from –60 to –75 mV by current steps. Amplitudes of voltage sag were measured at the end of the 400-ms steps. **(B)** Amplitudes of voltage sag of control (*n* = 15 cells) and mutant neurons (*n* = 13 cells) at P14-16. ^∗∗^*p* = 0.003, Wilcoxon test. **(C)** *I*_h_ activated using a 6-s step from –50 to –100 mV in a control (black) and mutant (red) neuron from a SERT-Cre; *Grin1^fl/fl^*; *Rosa*^*Ai*14^ at P16. The amplitude of *I*_h_ was measured at the end of the voltage step (indicated in the lines with arrows). **(D)** Amplitudes of *I*_h_ of control (*n* = 19 cells) and mutant neurons (*n* = 20 cells) from 16 male mice recorded at P14-16. ^∗∗∗^*p* < 0.001, Wilcoxon test. **(E)** Analysis of voltage-dependence of HCN channels. The top traces in blue show the voltage step protocol and lower traces in black are currents recorded in a control neuron at P15. The insert shows the tail currents (scale bar 200 ms). **(F)** I-V curves of steady-state *I*_h_ using the tail currents recorded from control (*n* = 12) and mutant (*n* = 11) neurons from 11 mice at P14–16. Data from control (black empty circles) or mutant (red dots) were fitted to a Boltzmann equation. *V*_h_, the membrane potential of half-maximal activation, was –88.5 ± 1.8 mV for control and –88.6 ± 3.8 mV for mutant cells (*NS*, *p* = 0.23, Wilcoxon test). **(G)** Effects of ZD7288 (20 μM, bath application) on firing patterns of a VPm neuron in a WT mouse at P16. **(H)** Rheobase of VPm neurons before and during ZD7288 application (^∗∗∗^*p* < 0.001, Wilcoxon test). **(I)** Slope of the F-I curve of VPm neurons before and during ZD7288 application (^∗∗∗^*p* < 0.001, Wilcoxon test). Data were from 14 neurons in 6 WT mice at P14-16.

Hyperpolarization-activated cation channels have important functions in the regulation of neuronal excitability and these channels are up-regulated during early life in many parts of the brain including the thalamus (Kanyshkova et al., 2009). We first analyzed voltage sag generated by *I*_h_ in VPm neurons. Control and mutant neurons were hyperpolarized by current steps from -60 to -75 mV (**Figure 6A**). Mutant neurons had smaller voltage sag than control neurons (**Figures 6A,B**), indicating a down-regulation of HCN channel function in mutant neurons.

Next, we analyzed *I*_h_ in VPm neurons using voltage clamp recording. Compared with control neurons, mutant neurons had much smaller *I*_h_ (**Figures 6C,D**). In contrast, *Grin1* deletion did not significantly affect the kinetics of *I*_h_ activation (720.5 ± 28.5 ms, *n* = 20 cells for mutant vs. 651 ± 27.8 ms, *n* = 19 cells for control, *p* = 0.074, Wilcoxon test) or inactivation (240.0 ± 5.8 ms vs. 232.2 ± 6.0 ms, *p* = 0.49, Wilcoxon test). To determine whether NMDAR deletion alters voltage dependency of HCN channels, we analyzed the current-voltage (I-V) curve for steady-state *I*_h_ using tail current measurement (**Figure 6E**). The I-V relationship of steady-state *I*_h_ was not significantly different between mutant and control neurons (**Figure 6F**). The density of *I*_h_, estimated using the maximal tail current, was significantly reduced in mutant neurons (7.9 ± 0.5 pA/pF, n = 17 cells for mutant vs. 9.7 ± 0.5 pA/pF, *n* = 15 cells for control, ^∗^*p* = 0.034, Wilcoxon test). Together, these results suggest that deletion of NMDARs reduces the number of HCN channels in thalamic neurons.

To determine whether HCN channels are implicated for the changes in intrinsic excitability in NMDAR-deficient neurons, we examined the effects of blocking HCN channels on excitability of VPm neurons in wild type (WT) mice. Bath application of the specific *I*_h_ blocker ZD 7288 (20 mM) significantly enhanced intrinsic excitability of these cells (**Figure 6G**). Similar to the effects of NMDAR deletion, the blockade of *I*_h_ reduced the rheobase of spiking and increased the slope of the F-I curve of WT neurons (**Figures 6H,I**). In the presence of ZD7288, the rheobase and slope of the F-I curve of WT neurons were comparable to those of GluN1-deficient neurons (0.562 ± 0.037 vs. 0.607 ± 0.048 Hz/pA for F-I slope, *p* = 0.76, Wilcoxon test; 69.2 ± 4.8 vs. 61.6 ± 8.4 pA for rheobase, *p* = 0.24, Wilcoxon test). The blockade of *I*_h_ in WT neurons also caused an increase in input resistance (109.5 ± 6.9 vs. 172.6 ± 8.2 MW, *n* = 14 cells; ^∗∗∗^*p* < 0.001, Wilcoxon test). These results suggest that NMDAR signaling regulates intrinsic excitability of thalamic neurons at least partially through *I*_h_.

### NMDA Receptors Regulate Intrinsic Excitability through mTOR Signaling

**FIGURE 7:**
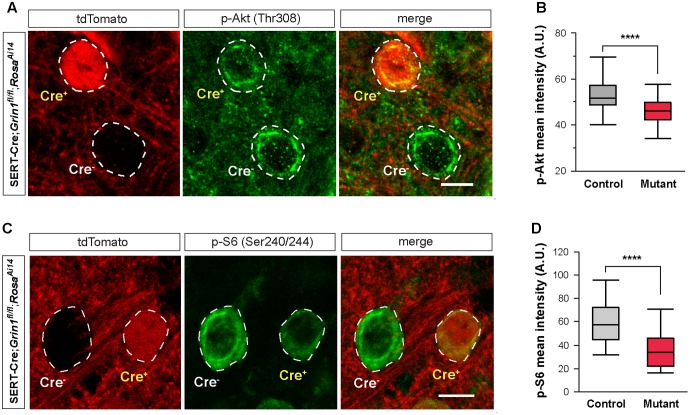
Deletion of NMDARs down regulates mTOR signaling in thalamic neurons. **(A)** Confocal images of thalamic sections immunostained for p-Akt (Thr308) from a SERT-Cre; *Grin1^fl/fl^*; *Rosa*^*Ai*14^ mouse at P18. Cell bodies are outlined with dash lines. Scale bar 10 μm. **(B)** Mean intensity of the p-Akt signal measured at cell body of control and *Grin1* mutant neurons (^∗∗∗∗^*p* < 0.0001, Wilcoxon test). Data were obtained from 90 control and 93 mutant cells from 3 mice at P18-19. **(C)** Confocal images of thalamic sections immunostained for p-S6 (Ser240/244) from a SERT-Cre; *Grin1^fl/fl^*; *Rosa*^*Ai*14^ mouse at P15. Scale bar 10 μm. **(D)** Mean intensity of the p-S6 signal measured at cell body of control and *Grin1* mutant neurons (^∗∗∗∗^*p* < 0.0001, Wilcoxon test). Data were obtained from 31 control and 33 mutant cells from 4 mice at P15–17.

Deletion of NMDARs disrupts many molecular pathways. Because of the reduction in cell size, we hypothesized that the mTOR pathway, known to play a critical role in cell growth (Hay and Sonenberg, 2004), is implicated in NMDAR-mediated regulation of intrinsic excitability. The PI3K/Akt pathway is upstream of mTOR activation. We first analyzed Akt phosphorylation in the VPm of SERT-Cre; *Grin1^fl/fl^*; *Rosa*^*Ai*14^ mice by measuring immunofluorescence of phosphorylated Akt (p-Akt) in single cells. We found that mutant neurons had significantly lower p-Akt signal than neighboring control neurons (**Figures 7A,B**). This result indicates that deletion of NMDARs down-regulated Akt function in thalamic neurons.

A key downstream effector of the mTOR signaling is the ribosomal protein S6. Activation of mTOR phosphorylates S6 through the S6 kinase (Hay and Sonenberg, 2004). We analyzed S6 phosphorylation in the VPm of SERT-Cre; *Grin1^fl/fl^*; *Rosa*^*Ai*14^ mice with immunofluorescence using an antibody against phosphorylated S6 (p-S6). Mutant neurons had much lower p-S6 signal than neighboring control neurons (**Figures 7C,D**). Together, our results show that deletion of NMDARs down-regulates Akt/mTOR signaling in VPm neurons.

**FIGURE 8:**
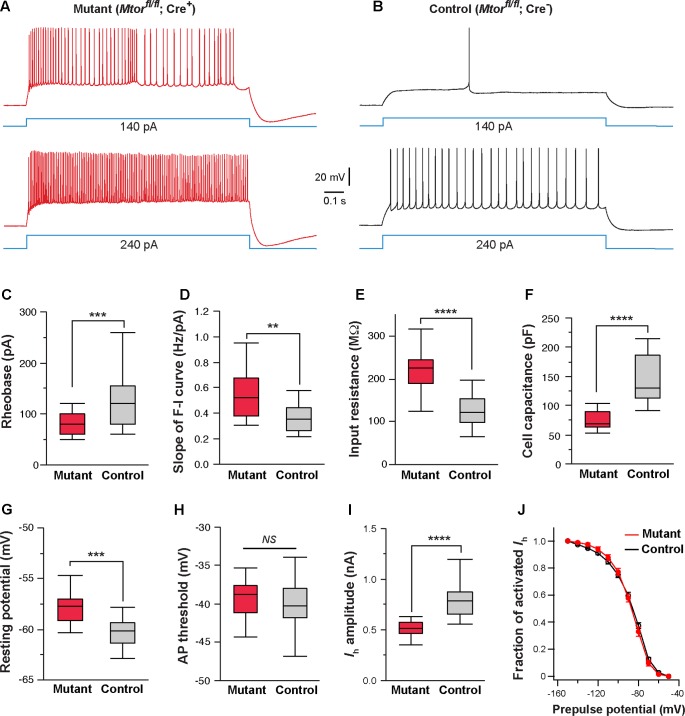
Mosaic deletion of *Mtor* in thalamic neurons recapitulates the effects of NMDAR deletion. **(A,B)** Firing patterns of an *Mtor* mutant neuron (*Mtor^fl/fl^*; Cre^+^) and a control (*Mtor^fl/fl^*; Cre^-^) neuron from a SERT-Cre; *Mtor^fl/fl^*; *Rosa*^*Ai*14^ mouse at P16. Membrane potential was adjusted to –60 mV before each current step. **(C)** Rheobase of mutant and control neurons (^∗∗∗^*p* < 0.001, Wilcoxon test). **(D)** Slope of the F-I curve of mutant and control neurons (^∗∗^*p* = 0.0011, Wilcoxon test). **(E)** Input resistance of mutant and control neurons (^∗∗∗∗^*p* < 0.0001, Wilcoxon test). **(F)** Whole-cell capacitance of mutant and control neurons (^∗∗∗∗^*p* < 0.0001, Wilcoxon test). **(G)** Resting potential of mutant and control neurons (^∗∗∗^*p* < 0.001, Wilcoxon test). **(H)** AP threshold of mutant and control neurons (*NS*, p = 0.28, Wilcoxon test). **(I)** Amplitude of *I*_h_ of control and mutant neurons (^∗∗∗∗^*p* < 0.0001, Wilcoxon test). Data presented in **(C)** through **(I)** were obtained from 22 mutant and 16 control neurons from 6 mice at P14-16. **(J)** I-V curves of steady-state *I*_h_ for control (*n* = 11) and *Mtor* mutant neurons (*n* = 11) from 5 mice. *V*_h_ was –87.6 ± 3.7 mV for control and –86.9 ± 3.3 mV for mutant cells (*NS*, *p* = 0.84, Wilcoxon test).

Next, we examined the effects of *Mtor* deletion on intrinsic excitability. We used the SERT-Cre and the conditional *Mtor* allele (Risson et al., 2009) to perform mosaic deletion of *Mtor* in the VPm, and analyzed intrinsic properties of mutant (*Mtor^fl/fl^*; Cre^+^) and control neurons (*Mtor^fl/fl^*; Cre^-^) in SERT-Cre; *Mtor^fl/fl^*; *Rosa*^*Ai*14^ mice aged P14-16. Deletion of *Mtor* mimicked the effects of NMDAR deletion in VPm neurons (**Figures 8A–J**). Mutant neurons showed hyperexcitability with lower rheobase (**Figure 8C**), steeper slope of the F-I curve (**Figure 8D**), higher input resistance (**Figure 8E**), and lower whole-cell capacitance (**Figure 8F**). Mutant neurons deficient also exhibited a more depolarized resting potential than control neuron (**Figure 8G**) but no change in AP threshold (**Figure 8H**). As in the case of NMDAR-deficient neurons, neurons deficient in mTOR had smaller *I*_h_ (**Figure 8I**), but no change in the I-V relationship of *I*_h_ activation (**Figure 8J**). We also analyzed changes in cell size in fixed sections from SERT-Cre; *Mtor^fl/fl^*; *Rosa*^*Ai*14^ mice at P17. Soma areas of mutant neurons were significantly smaller than those of control neurons (196.9 ± 2.9 μm^2^, *n* = 103 vs. 249.6 ± 5.6 μm^2^, *n* = 66; ^∗∗∗∗^*p* < 0.0001, Wilcoxon test). To examine the possibility that the deletion of *Mtor* down-regulates NMDARs, we analyzed NMDAR-mediated EPSCs in VPm neurons in SERT-Cre; *Mtor^fl/fl^*; *Rosa*^*Ai*14^ mice aged P15-16. There was no difference between mutant and control neurons in NMDAR-mediated EPSCs (Supplementary Figure S2), indicating that the deletion of *Mtor* does not alter NMDAR function in these cells. Together, these results support the role of mTOR signaling in NMDAR-mediated regulation of intrinsic excitability.

## Discussion

Previous studies have demonstrated that NMDARs have important functions in the development of dendrites and synapses. Here we provide genetic evidence that NMDARs are required for the development of intrinsic excitability of neurons. Mosaic deletion of *Grin1* in thalamic neurons causes hyperexcitability and a reduction of cell size through cell-autonomous mechanisms. Our results suggest that NMDAR-mediated signaling is a key mechanism controlling the maturation of neurons.

Neuronal activity plays an important role in the maturation of intrinsic properties during development (Spitzer, 2006). As the predominant glutamatergic receptor in neonatal brain, NMDARs have essential functions in the regulation of activity of neuronal circuits during development. However, the role of NMDARs in the maturation of intrinsic neuronal excitability is not well understood. Single-cell deletion of all AMPARs and NMDARs in the hippocampus of newborn mice caused hyperexcitation of CA1 pyramidal neurons (Lu et al., 2013), but it was unknown whether the effect was due to the lack of AMPARs or NMDARs. Deletion of NMDARs from most of the excitatory neurons in the forebrain using a *Camk2a*-Cre also led to increased intrinsic excitability of pyramidal neurons in adult mice (Tatard-Leitman et al., 2015). However, because of the late onset of the *Camk2a-Cre* expression (Tsien et al., 1996), the deletion of NMDARs in these mice probably occurs after 3 weeks of age, when the intrinsic properties of pyramidal neurons have reached the mature level. Furthermore, the *Camk2a* conditional *Grin1* knockout mice also showed significant increases in cortical excitatory synaptic transmission and network activity, which could indirectly affect neuronal excitability. By using mosaic *Grin1* deletion in the thalamus during early postnatal development, we find that ablation of NMDARs leads to hyperexcitability in mutant neurons without any effect on neighboring control neurons. Our results provide the first genetic evidence for cell-autonomous functions of NMDARs in the regulation of intrinsic neuronal excitability.

Intrinsic excitability of the neuron is determined by its morphology and the expression of various ion channels. Previous studies using single-cell gene deletion have shown either no or subtle effect of NMDAR deletion on neuronal morphology. Single-cell deletion of *Grin1* from CA1 pyramidal neurons in the hippocampus had no effect on dendritic length or branch points (Adesnik et al., 2008). Mosaic deletion of *Grin2B*, the predominant GluN2 subunit in the developing brain, disrupted dendrite patterning of granule neurons in the dentate gyrus and spiny stellar neurons in cortical layer 4 (Espinosa et al., 2009). Neurons without *Grin2b* had more primary dendrites than control neurons although the total length of dendrites and branch numbers did not change. Increased dendritic branching was also observed in cortical layer 4 neurons in mice with cortex-restricted deletion of *Grin1* (Datwani et al., 2002). It was unclear in these studies whether cell body size was affected by NMDAR deletion. Together, these previous results suggest that NMDARs have important functions in dendrite patterning but is not required for the growth of dendrites in these neurons.

In contrast, our results show that NMDARs are required for neuronal growth in thalamic neurons. VPm neurons deficient of NMDARs have smaller cell body size, fewer dendritic branches, and shorter dendrites. The reduction in cell body size is consistent with our findings of higher input resistance in mutant neurons. Deletion of *Grin1* in cortical pyramidal neurons had no effect on input resistance (Tatard-Leitman et al., 2015), indicating that the morphology of these cells was not significantly affected by NMDAR deletion. On the other hand, selective deletion of *Grin1* from GABAergic neurons in the cortex disrupted dendrite growth in GABAergic interneurons expressing Reelin (De Marco Garcia et al., 2015). Thus, the function of NMDARs in the development of neuronal morphology is likely to be cell-type specific.

Several studies have shown that HCN channels have important roles in activity-dependent regulation of intrinsic excitability in the brain. Theta-burst stimulation of hippocampal pyramidal neurons, which induced long-term potentiation, also led to a persist decrease of intrinsic excitability (Fan et al., 2005). This reduction in intrinsic excitability required NMDAR activation and was mediated through upregulation of *I*_h_. HCN channels are highly expressed in dendrites (Tsay et al., 2007; Huang et al., 2009; Vaidya and Johnston, 2013). Sensory deprivation caused dendritic hyperexcitability in layer 5 pyramidal neurons of the somatosensory cortex and the effect was associated with a decrease of *I*_h_ (Breton and Stuart, 2009), although whether this effect requires NMDARs is unknown. We find that deletion of NMDARs in thalamic neurons causes a decrease of *I*_h_ and pharmacological block of *I*_h_ in wild type neurons mimics the effects of NMDAR deletion on intrinsic excitability. Our results support a role of NMDARs in activity-dependent regulation of HCN channel function and intrinsic excitability.

The exact molecular mechanisms by which NMDARs regulate neuronal morphology and intrinsic excitability are not well understood. Previous studies have shown that NMDAR activation is required in activity-dependent up-regulation of Akt/mTOR signaling in hippocampal neurons (Cammalleri et al., 2003; Gong et al., 2006; Pirbhoy et al., 2016). We find that thalamic neurons deficient of NMDARs have reduced levels of p-Akt and p-S6, indicating that deletion of NMDARs causes a down-regulation of Akt/mTOR signaling in these neurons. Supporting the role of mTOR signaling, we find that mosaic deletion of *Mtor* from the thalamus recapitulates the effects of NMDAR deletion on intrinsic excitability and cell body size. Our results suggest that a major cellular function of NMDARs during development is to regulate protein synthesis in activity-dependent manner through mTOR signaling.

Several recent studies have shown that abnormal up-regulation of mTOR signaling during development affects morphology and excitability of neurons. Tuberous sclerosis complex 1 (*Tsc1*) suppresses mTOR signaling, and loss of function mutations of *Tsc1* cause tumors and a variety of neurological symptoms including intellectual disability, seizures, and autism (Caban et al., 2017). In the mouse, single-cell deletion of *Tsc1* in the neocortex during embryonic development increased the level of p-S6 and soma size in the affected neurons (Feliciano et al., 2011). Increased soma size was also observed in the thalamus using mosaic deletion of *Tsc1* (Normand et al., 2013). Deletion of *Tsc1* at E12.5 in thalamic neurons reduced input resistance and the slope of the F-I curve (Normand et al., 2013), indicating that hyperactivity of mTOR signaling during embryonic development leads to hypo-excitability. We find that deletion of *Mtor* in thalamic neurons during development leads to opposite changes in membrane properties and cell firing. Our results demonstrate that mTOR is required for the development of neuronal excitability.

Mutations of *GRIN1* have been reported in patients with neurodevelopmental disorders including early onset epilepsy, severe intellectual disability, motor dysfunction, and generalized cerebral atrophy (Ohba et al., 2015; Lemke et al., 2016). Although the mutations are predicted to disrupt NMDAR function, the pathophysiology of the diseases is unknown. Our results suggest that in addition to synaptic dysfunction, changes in neuronal morphology and intrinsic excitability may have important roles. Future studies are needed to analyze the effects of human *GRIN1* mutations on function of neurons and neuronal circuits.

## Author Contributions

Z-WZ and GH designed research. GH performed research. GH and Z-WZ analyzed data. Z-WZ wrote the paper.

## Conflict of Interest Statement

The authors declare that the research was conducted in the absence of any commercial or financial relationships that could be construed as a potential conflict of interest.
