# Supplementary material for: NMDA Receptors Regulate the Development of Neuronal Intrinsic Excitability through Cell-Autonomous Mechanisms
Source: Front Cell Neurosci. 2017 Nov 7;11:353. doi: 10.3389/fncel.2017.00353 (PMC5674002; doi:10.3389/fncel.2017.00353)
Supplement: Supplementary file 1 [file Presentation_1.PDF]

Supplementary Materials

NMDA receptors regulate the development of neuronal intrinsic excitability through cell-autonomous mechanisms

Guoqiang Hou and Zhong-Wei Zhang

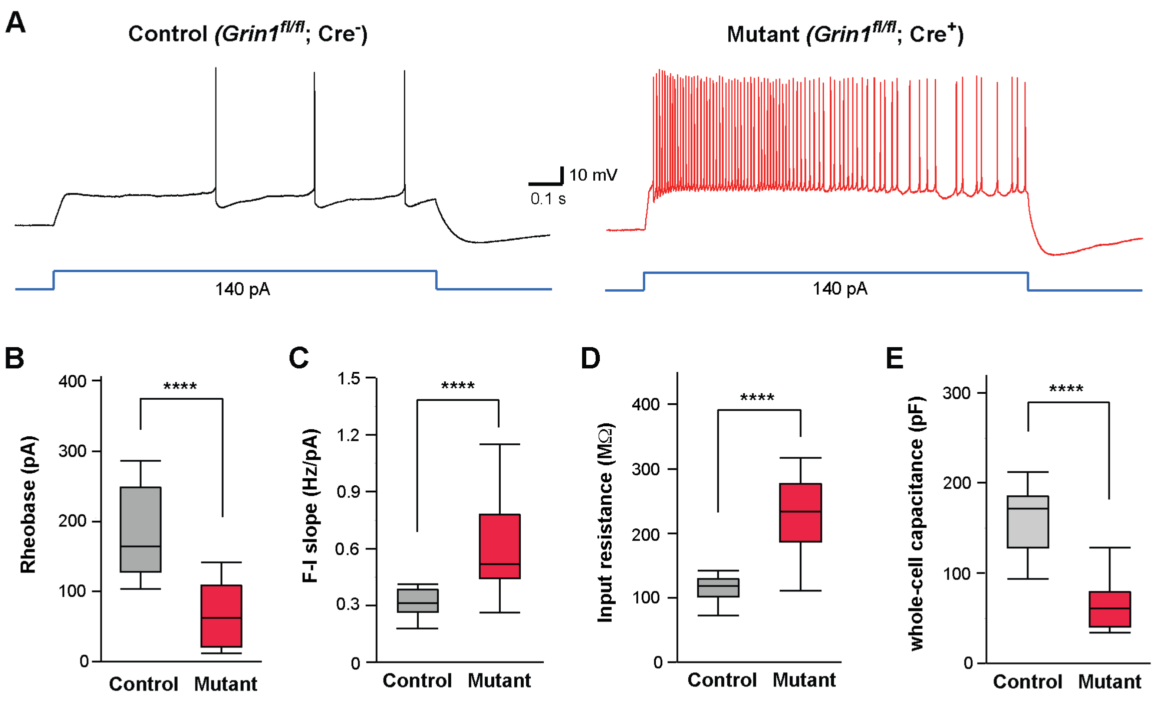

Supplementary Figure S1. Effects of NMDAR deletion on intrinsic excitability are not affected by the presence of synaptic blockers. (A) Firing patterns of a *Grin1* mutant (*Grin1*$^{fl/fl}$; Cre^{+}, traces in red on the left) and a control (*Grin1*$^{fl/fl}$; Cre^{-}, traces in black on the right) neuron recorded in the presence of picrotoxin (100 µM), DNQX (10 µM), and kynurenic acid (1 mM) at P15 from a SERT-Cre; *Grin1*$^{fl/fl}$; *Rosa*^{Ai14} mouse. (B) Rheobase (**** $p < 0.0001$, Wilcoxon test), (C) Slope of the F-I curve (**** $p < 0.0001$, Wilcoxon test), (D) Input resistance (**** $p < 0.0001$, Wilcoxon test), and (E) Whole-cell capacitance (**** $p < 0.0001$, Wilcoxon test) of mutant and control neurons. Data were collected from 19 mutant and 16 control cells from 4 mice at P14-16.

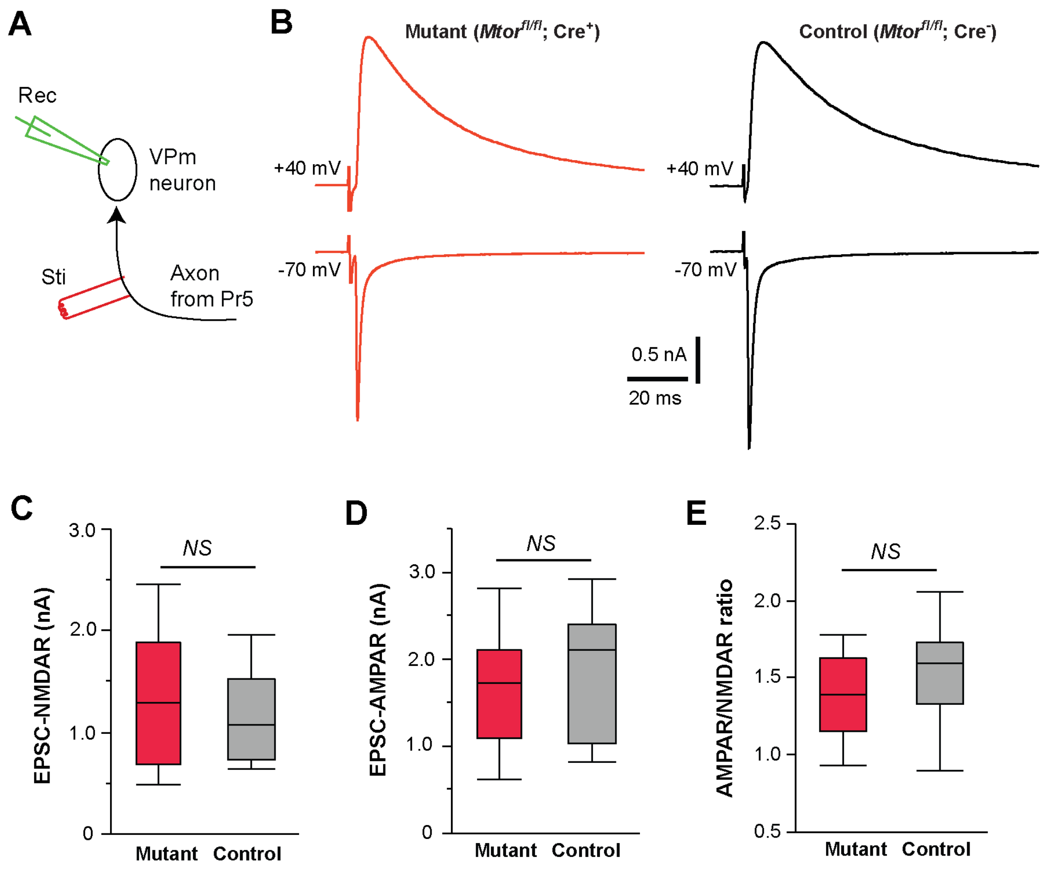

Supplementary Figure S2. Deletion of *Mtor* does not alter NMDAR function at the thalamic relay synapse. (A) Diagram showing the method of EPSC recording. EPSCs were recorded in VPm neurons in SERT-Cre; $Mtor^{fl/fl}$; $Rosa^{Ai14}$ mice at P15-16. A bipolar electrode (in red) was placed in the medial lemniscus to stimulate axons from the principal trigeminal nucleus (Pr5). (B) The maximal EPSCs at holding potentials of +40 and -70 mV recorded from a mutant (traces in red) and a control (traces in black) neuron at P16. (C) Amplitudes of NMDAR-mediated EPSCs (*NS*, p = 0.84, Wilcoxon test). The amplitude of NMDAR-mediated EPSCs was measured at 10 ms from the beginning of EPSCs recorded at +40 mV. (D) Amplitude of AMPAR-mediated EPSCs (*NS*, p = 0.68, Wilcoxon test), and (E) the AMPAR/NMDAR ratio of mutant and controls neurons (*NS*, p = 0.11, Wilcoxon test). Data were obtained from 16 mutant and 17 control cells from 8 mice.
